# Best Interest, Basic Interest, and Parental Autonomy in Child Vaccination Choices. On Roland Pierik and Marcel Verweij’s *Inducing Immunity?*

**DOI:** 10.1093/phe/phag009

**Published:** 2026-07-13

**Authors:** Alberto Giubilini

## Abstract

Roland Pierik and Marcel Verweij, in their excellent book Inducing Immunity?, draw a distinction between ‘best interest’ and ‘basic interest’ and use such distinction as a criterion for legitimate state interference with parental choices, including in the case of vaccination. While protection of children’s basic interests does provide a solid basis for the justification of state intervention in vaccination decisions, I want to offer some considerations and raise some questions about how the distinction between best and basic interest is conceptualized in the book, about some assumption on the relationship between freedom of choice and best interest that that distinction implies, and about some broader implications for discussion of child vaccination. I will conclude by raising a question about the justification for mandating vaccination within Pierik’s and Verweij’s ethical framework.

In their excellent book *Inducing Immunity?*, Roland Pierik and Marcel Verweij (2024) suggest that parents should be free to make choices that they consider to be in their children’s best interest, except when such choices undermine or threaten children’s ‘basic interests’. The latter are typically protected by legislations, Court rulings, and international charts of rights. The textbook example which also Pierik and Verweij refer to is that of Jehovah’s witnesses who refuse blood transfusion for their children, and whose choice is typically overruled by courts. Parents should be free to raise their children according to the requirements of their religion, but only as long as that doesn’t threaten their children’s *basic* interest in basic medical care. Solid ethical arguments and legal framework support this approach, including rulings from the US Supreme Court and the European Court of Human Rights. So far so good.

Enter vaccination. Pierik and Verweij suggest vaccination should be treated in the same way when assessing the legitimate sphere of state interference with parental choices. That is, if vaccinating one’s child protects a child’s basic interests, then parental refusal to vaccinate may permissibly be overridden. If, however, vaccinating one’s child merely protects a child’s *best* interest as determined by the parents, then parental freedom of choice should be respected.

Here, I would like to offer some brief observations about how that distinction is drawn in Pierik’s and Verweij’s book, about some assumption around the relationship between freedom of choice and best interest that that view implies, and about some broader implications for discussion of child vaccination. I will conclude by raising a question about the justification for state enforcement of child vaccination within Pierik’s and Verweij’s ethical framework.

## Best Interest and Parental Freedom

The authors notice that the term ‘best interest’ can be disambiguated between two terms: ‘what is best for the child, as determined by the parents’ and ‘basic interest, which is ultimately a matter of government responsibility’ (Pierik and Verweij, 2024: 88). It seems that the disambiguation is really between a subjective and– for lack of a better word—a ‘non-subjective’ notion of what counts as in a child’s interest, where basic interests belong to the second group. Pierik and Verweij provide a plausible list of the latter type: access to clean water and nutrition, protection against lethal and disabling diseases, some level of safety, some basic education.

I am hesitant to call the non-subjective notion of interest an ‘objective’ notion, as objectivity might be taken to rule out some degree of context-sensitivity of what counts as an interest or a basic interest, and I am not sure that context-sensitivity can be ruled out. Perhaps what counts as basic interest is better described as ‘intersubjectively agreed upon’, or as ‘reasonably considered’ basic interest, rather than objectively basic interests. To avoid unnecessary philosophical complications with this terminology, I will stick with the negative terminology of ‘non-subjective’ notion of interest and of basic interest.

The problem is that this disambiguation doesn’t seem to leave room for a notion of best interest that is non-subjective, but at the same time distinct from that of basic interest. Yet, this is a category that, I suggest, we might want to preserve, not least because some child vaccinations might well belong to it. For example, a balanced diet including meat would be in the best interest of a child from a nutritional point of view even when parents think it is in the best interest of their children—that is, it is ‘best for the child, as determined by the parents’- to be raised vegetarian. At the same time, however, a vegetarian diet doesn’t necessarily violate a basic interest of the child, at least when sufficient attention is paid to relevant nutrients. It’s somewhere in between: not ideal, but good enough, at least when done properly. Or suppose parents choose to send their children to a good but not outstanding school, because that better fits the parents’ work schedule or because that is more consistent with the parents’ own worldviews (say, a religious school), although the outstanding school would have been a reasonable option. It seems that although parents are not acting in the child’s *best* interest, they are not violating the child’s basic interest in minimal education either. Again, they are doing something in between, which is good enough.

These examples seem to suggest that a notion of ‘best interest’ that is independent of parental choices exists and is distinct both from the non-subjective notion of basic interest and from the subjective notion of ‘best interest’ as conceptualized by Pierik and Verweij. It seems that the notion of ‘what is best for the child, as determined by the parents’ would sometimes occupy some middle, ‘good enough’ ground, rather than belonging to the realm of ‘best interest’ as we know it. Of course, the two can overlap. In fact, the authors argue that they typically do, since parents’ daily interactions with their children make them well suited to know what would be more beneficial for their children (Pierik and Verweij, 2024: 92). However, if all this is correct, the legitimate space of parental autonomy and freedom of choice doesn’t *necessarily* overlap and is logically independent of the ‘best interest’ of their children.

That freedom and autonomy might well be worth protecting from state interference, but if so, it’s not because they protect children’s *best* interest. As we have seen, it often does not. Rather, it is because parental autonomy and freedom of choice matter for their own sake. If they do, then parents should be allowed to make suboptimal choices about their children, at least as long as these don’t threaten basic interests, as Pierik and Verweij rightly argue.

Pierik and Verweij might be adopting the subjectivist interpretation of best interest because they seem committed to two claims at the same time. One is that parents have a moral obligation to pursue their children’s best interest, as many legal documents and international codes prescribe.^1^ The other is the centrality of parental autonomy and the importance of leaving some room for parental discretion, arguing that the state should not intervene in determining a child’s ‘best interest’. Thus, it seems they offer an interpretation of the notion of ‘best interest’ that makes it consistent with parental autonomy *by definition*—‘what is best for the child, as determined by the parents’.

However, it is questionable that parents have a moral obligation to pursue their children’s *best* interest (in a non-subjective sense). In fact, it is also questionable that parents know best what such interest is, a claim which Pierik and Verweij use to support the view that parental autonomy is likely to promote children’s best interest. These claims are only true in a limited set of cases. Therefore, there seems to be no need to postulate that subjectivist understanding of ‘best interest’ that would enable us to uphold both. Let’s consider both claims briefly.

First, arguably, parents do not have a moral obligation to pursue what is *best* for a child, and not even a moral obligation to do what is best among reasonably available options. ‘The best’ is a very high, indeed the highest possible, bar. The problem with setting the bar that high is that meeting that standard would be overly demanding and therefore, on most understandings of morality, beyond the call of moral duty.

Consider again parents who send their children to a good but not outstanding school, or that don’t have their child take piano lessons. Or perhaps parents who only do either of these two things, when both would be feasible if parents were prepared to sacrifice more of their time and money. Arguably, such parents don’t necessarily fall short of their ethical obligations, even if they are not doing what would be *best* for their children.

The notion of ‘best interest’ and an obligation to act in a patient’s best interest might retain their centrality as criteria for medical decisions within a medical law and bioethical paradigm, where there typically are a limited set of clinical options all reasonably available. It is relatively easy, or at least medical criteria exist, to identify which option is in someone’s best interest. Often in such contexts acting in someone’s best interest makes a significant difference for the future quality of life of that individual, and indeed sometimes a difference between life and death. For all these reasons, other things being equal, in such contexts the best interest of a child ought to be prioritized. The case of Jehovah’s witnesses mentioned above is the paradigmatic example. However, when options for a child are discussed outside of a clinical context and concern lifestyle, education, nutrition, upbringing, and so on, it is hard to ground parental obligations in a notion of children’s best interest, as obvious counterexamples of parents permissibly not pursuing a child’s best interest can easily be found.

Second, parents do not always know what is in their children’s best interest or how to act to promote it—indeed, I would suggest, they often do not. And, even when they do, they don’t always act upon it. In many cases, that is fine and should be less worrying than it might sound, as chances are in most cases parental decisions would anyway be ‘good enough’, to recall the category introduced above. There are two types of cases that are relevant to this discussion: cases of genuine epistemic uncertainty around best interest and cases where parents fail to act upon their children’s best interest, regardless of whether they are in the position to identify it.

About the first type, for instance, it’s difficult to establish whether and when vaccinating one’s children against COVID-19 is in their best interest, given the uncertainty and disagreement around the matter even among experts (Giubilini et al, 2025). Depending on individual circumstances and disease profiles, other vaccines might be like this at least for some children.

And about the second type, oftentimes vaccines are *clearly* in a child’s *best* interest—with no plausible epistemic uncertainty—and parents not vaccinating their children are falling short of acting in a child’s best interest. There are different reasons why they might do that. For instance, they might have incorrect views about effectiveness and safety of vaccines; or they might have correct views about those aspects, but have other reasons—cultural, religious, philosophical—not to vaccinate; or they might have correct views and no countervailing reason, but face access barriers that, rightly or wrongly, they consider too difficult to overcome. And so on.

Falling short of acting in a child’s best interest, including refusing beneficial vaccination for them, for any such reason might well be ethically acceptable and it might well be something the state should not interfere with. But when that is the case, it is important to be clear on the reason why. And the reason is respect for parental freedom or parental autonomy,^2^ rather than the claim that parents always know best what is in a child’s best interest. For often they do not.

## A Child’s Interests and Vaccination: Concluding Thoughts

From what I have argued, it seems there are rather three, not two, relevant types of interests that children have, according to the kind of relevance that Pierik and Verweij focus on. Some are ‘best interest’ in a non-subjective sense. Others occupy a middle ground—something that is not a child’s best interest, but it’s not detrimental to children, either. And some are basic interest in the non-subjective sense Pierik and Verweij describe them.

The point we have just reached is relevant for the discussion of child vaccination and parents’ vaccination choices for their children. It seems we should reject a claim that a beneficial vaccine—that is, a vaccine whose benefits outweigh the risks, given the circumstances—is either a basic interest or something that is in a child’s best interest only as long as parents consider it to be, and *tertium non datum*. Yet, this is precisely what Pierik and Verweij’s subjective notion of best interest, as separate from basic interest, seems to imply.

Pierik and Verweij′s disambiguation presented above prevents us from saying that certain child vaccinations are in a child’s best interest, in the non-subjective sense of the term (and so regardless of whether parents’ think they are), even if they are not necessarily a basic interest. Yet, that such vaccinations are a non-subjective best interest, but not a basic interest, is a possibility and a conceptual space that we might want to preserve. In fact, depending on circumstances, sometimes routine child vaccinations as included in the vaccination schedule of many countries seem to be precisely like that. It’s in children’s best interest to receive them, but not receiving them doesn’t necessarily violate a basic interest. Chickenpox vaccination, for instance, which some countries include in their routine vaccine schedule for children, is a good candidate for this category. Parents who fail to vaccinate their child against chickenpox are arguably not acting in their child’s best interest, although a case can be made for why they should nonetheless be free to refuse that vaccine for their children. That is, given the nature of chickenpox, this would not threaten a child’s *basic* interest. However, the authors’ interpretation and disambiguation of the notion of ‘best interest’ doesn’t seem to allow to say that. Rather, it would require us to say that parents not vaccinating their children against chickenpox are merely doing ‘what is best for the child as determined by parents’, which is not the same as acting in a child’s best interest.

Pierik and Verweij are right, I think, in identifying in the protection of children’s basic interest a necessary condition for the justification of state interference with parental vaccination choices. But I don’t think that arguing that needs to come at the cost of denying that oftentimes vaccinating children is in children’s best interest in the non-subjective sense of the term, and I am not sure Pierik and Verweij’s conceptual framework manage to avoid that implication.

## A Child’s Interests and Vaccination: a Question

Whether and when childhood vaccination, as well as being in a child’s best interest in the non-subjective sense, also protects the child’s *basic* interest is a more complex question. Measles vaccination for instance seems to be one such case. The authors say that while common childhood vaccinations do protect children’s basic interests (Pierik and Verweij, 2024: 97), this doesn’t imply that such immunizations can be imposed against parents’ will. Instead, they say that such imposition is only ethically justifiable when ‘immunization offers the only genuinely effective protection against an imminent and serious threat—namely, when an unvaccinated child has been exposed to a potentially lethal or disabling pathogen such as measles or hepatitis B’ (Pierik and Verweij, 2024: 97–98).

But wouldn’t this contradict the principle they repeatedly invoke, for example when they say that the ‘child’s basic interests, including the interest to be free from preventable diseases, […] define a harm threshold that sets limits on the freedom of parents to raise their children according to their conception of a good life’ (Pierik and Verweij, 2024: 94)? The question arises because when an imminent and serious threat presents itself, it might be too late to guarantee that freedom via vaccination—in fact, that there is an outbreak very likely means many got infected before being able to be effectively immunized. Consider, for instance, that it takes 2 weeks after measles vaccination for the vaccine to start providing sufficient immunity. Or is the assumption here that protection of basic interests is only a necessary, but not a sufficient condition for state intervention in vaccination decisions?

Or perhaps the authors, when they refer to protection of basic interests, are setting the conditions for some extreme measure like forced vaccination, as opposed to mere vaccine mandates? With forced vaccination, children would be vaccinated by some state authority, or by healthcare professionals on state authorities’ orders. This would seem more justifiable in the case of imminent and serious threats than in normal times, of course. But the same question as before arises: wouldn’t vaccination be required to protect basic interests also in normal times, since waiting until the emergency comes might be too late, and wouldn’t the authors’ ethical framework have implications that would be difficult to accept, such that forced vaccination of children would be justified also in normal times?

## Footnotes

1 For instance, the UN Convention on the Rights of the Child states that ‘[p]arents or, as the case may be, legal guardians, have the primary responsibility for the upbringing and development of the child. The best interests of the child will be their basic concern’ (Unicef, 1990).

2 A distinction I don’t have the space to get into here, but that for the purpose of this discussion is not relevant.
